# Differential diagnosis and clinical management of isolated prolonged activated partial thromboplastin time in a patient with Hashimoto thyroiditis-associated thyroid cancer: A case report

**DOI:** 10.1097/MD.0000000000026608

**Published:** 2021-07-09

**Authors:** Yuhong Zhong, Biyong Yan, Zhongqi Yu, Lin Wang

**Affiliations:** a Department of Clinical Hematology Laboratory, the Second Affiliated Hospital, Zhejiang University School of Medicine, Hangzhou, Zhejiang, PR China; b School of Medical Laboratory, Hangzhou Medical College, Hangzhou, Zhejiang, PR China; c Department of Emergency Medicine, Laboratory of Emergency Medicine, the Second Affiliated Hospital, Zhejiang University School of Medicine, Hangzhou, Zhejiang, PR China.

**Keywords:** activated partial thromboplastin time, Hashimoto thyroiditis, isolated prolongation, lupus anticoagulant, mixing studies

## Abstract

**Rationale:**

Patients preparing for surgery may have isolated, prolonged activated partial thromboplastin time (APTT). Cause analysis is warranted in patients who had neither bleeding symptom nor thromboembolic events because isolated prolongation of APTT may lead to unnecessary delayed surgical intervention or invasive procedure, even ineffective plasma infusion treatments. Here, we report a case of Hashimoto thyroiditis-associated thyroid cancer whose APTT was isolated prolonged and discuss the challenges of diagnosis and clinical management of this patient.

**Patient concerns:**

A 57-year-old woman was admitted to the hospital due to thyroid cancer. Anticoagulant assay was performed for this patient before surgery, she had normal values for prothrombin time, thrombin time, and fibrinogen, but had isolated prolonged APTT value (20 seconds longer than normal). However, the routine laboratory of the local hospital showed normal APTT and she did not have any abnormal bleeding or thrombotic episodes. Lupus anticoagulant (LA) was strongly positive according to mixing studies and modified dilute Russell viper venom time method, it was responsible for prolonged APTT.

**Diagnoses:**

Hashimoto thyroiditis-associated thyroid cancer whose APTT was isolated prolonged.

**Interventions:**

The isolated prolongation of APTT in this patient was due to LA. She had no history of anticoagulant medications and no spontaneous bleeding episodes. There should be no specific intervention before thyroidectomy.

**Outcomes:**

This thyroid cancer patient had an uneventful surgery and was discharged after a week.

**Lessons:**

Prolonged APTT is not considered an absolute indication for plasma infusion therapy in patients with LA. The correct identification of the cause of APTT prolongation is essential for proper treatment of the individuals.

## 1 Introduction

Activated partial thromboplastin time (APTT), as a routine preoperative coagulation test, is mainly used to evaluate the function of intrinsic coagulation pathway. It reflects various coagulation disorders and plays an important role in the diagnosis of hemophilia and disseminated intravascular coagulation, and in the evaluation of the safety and efficacy of unfractionated heparin.^[1]^

Generally, bleeding can be monitored by APTT before surgery. However, not all causes of prolonged APTT contribute to increased bleeding.^[2]^ Any patient with isolated spontaneous prolonged APTT should be further investigated to distinguish anticoagulants from factor deficiencies and lupus anticoagulant (LA). The incidence of thrombosis is higher than that of bleeding if the prolongation of APTT is due to the lack of contact factors or the presence of LA.^[3]^ Misinterpretation of isolated prolongation of APTT may result in unnecessary or even erroneous treatment, laboratory specialist and clinician should intensify investigations. Here, we reported a case of Hashimoto thyroiditis-associated thyroid cancer whose APTT was isolated prolonged.

## 2 Case presentation

### 2.1 Source of medical history and experimental methods

This study was approved by the Ethics Committee of the Second Affiliated Hospital of Zhejiang University School of Medicine. Written informed consent for publication of the details was obtained from the patient.

Clinical information was collected from the medical record, including medical history, exposure history, symptoms, laboratory findings, CT scans, B-mode ultrasonography, and clinical management.

Platelet count was measured at the Department of Clinical Laboratory using the XN9000 automatic hematology analyzer (Sysmex Corporation, Kobe, Tokyo, Japan), which is based on laser electrical impedance method; prothrombin time (PT), activated partial thromboplastin time (APTT), thrombin time (TT), fibrinogen were detected by a STA-R MAX analyzer (Diagnostica Stago, Saint-Denis, France) based on the Clauss method. All reagents used in this experiment were of matching ones. The specific detection steps were carried out in accordance with the manufacturer's instructions.

Mixing studies were performed using 2 types^[4,5]^: ① Immediate mixing study: patient plasma (C) was mixed (A) with an equal volume of pooled normal plasma (B), and then APTTs were immediately detected. The following formula was used: index of circulating anticoagulant (ICA) = (A–B) × 100/C. Two calculated thresholds were employed: ICA >15% was considered corrected and ICA <12% was not corrected; ② Time incubation mixing study: (a) mixed plasma was measured for APTT after incubation at 37 °C for 1 to 2 hours; (b) patient plasma and pooled normal plasma were first incubated separately, then mixed with equal volumes and APTT was measured. LA was present in the patient plasma when (a) − (b) <3 seconds, otherwise the presence of coagulation factor inhibitors was considered.

LA was detected with the modified dilute Russell viper venom time (dRVVT) method using ACL Top 700 hemostasis analyzer (Instrumentation Laboratory, Milan, Italy). The normalized ratio (dRVVT patient screen/dRVVT patient confirm)/(dRVVT screen control/dRVVT control confirm) was calculated, value ≥1.2 was considered positive. Anti-cardiolipin antibodies and Anti-β2 glycoprotein I antibodies were detected using ELISA assay (EUROIMMUN, Lübeck, Germany), values of >20 were considered positive for both.

### 2.2 Medical history and laboratory findings

A 57-year-old woman, was admitted to our hospital in November 2020 due to thyroid cancer. She was diagnosed with Hashimoto thyroiditis in 1995 and has been treated with levothyroxine tablets. She denied any other drug use, all other past medical or surgical history, and history of blood transfusion. She denied genetic or family history of cancer. Ultrasound examination of the thyroid gland found multiple bilateral thyroid nodules, enhanced CT and biopsy of the thyroid gland indicated bilateral thyroid papillary carcinoma. Routine laboratory tests were performed after admission, including routine hematology, biochemistry screen, and thyroid function tests. The patient had normal thyroid hormone levels (free triiodothyronine; free tetraiodothyronine; thyroid-stimulating hormone), but antithyroglobulin antibodies and thyroid peroxidase antibodies were well outside the normal range, whereas thyroglobulin was undetectable (Table 1). All these findings gave a diagnosis of Hashimoto thyroiditis combined with thyroid cancer, the patient underwent bilateral thyroidectomy.

**Table 1:** Results of coagulation parameters at local hospital and our hospital.

		Our hospital		
Indicators (Unit)/Test	Local hospital	Presurgery	Postsurgery	Reference value/Judgment criteria
PT, s	12.7	12.0	13.0	12.0–14.0
APTT, s	37.8	65.3	64.1	30.0–45.0
TT, s	17.2	18.6	16.5	<20.0
FIB, g/L	4.65	5.42	5.13	2.0–4.0
APTT immediate mixing study	—	25.0	23.5	ICA >15%: not corrected; ICA <12%: corrected
APTT time incubation mixing study	—	1.6 s extension	2.1 s extension	<3 s extension: LA; >3 s extension: factor inhibitors

APTT = activated partial thromboplastin time, FIB = fibrinogen, ICA = index of circulating anticoagulant, LA = lupus anticoagulant, PT = prothrombin time, TT = thrombin time.

**Figure 1:**
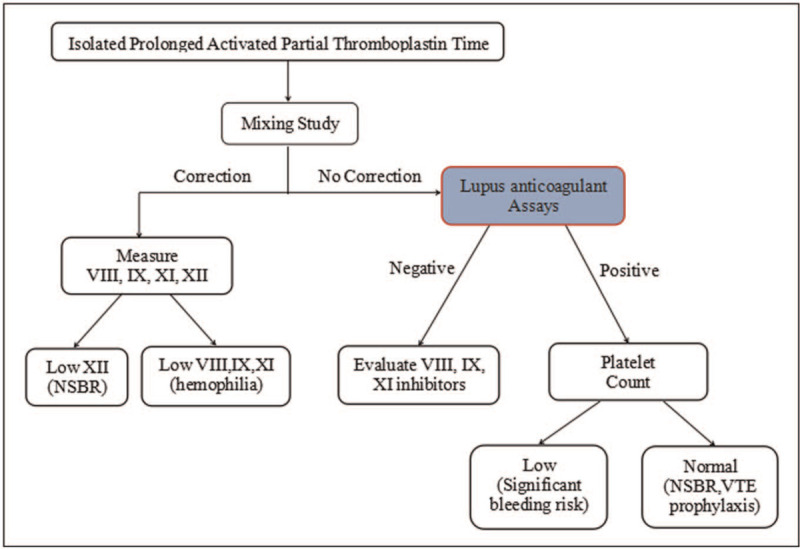
Diagnostic flowchart of isolated prolonged APTT. If the mixing study “correction,” measure factors VIII, IX, XI, and XII. A deficiency of FVIII, IX, or XI in addition to FXII are risk factors for bleeding. No correction of mixing study indicates the presence of inhibitors. A positive lupus anticoagulant accompanied by a low platelet count indicates the bleeding risk, otherwise, a thrombophilia evaluation should be performed. It is highly likely that there are specific inhibitors of factor VIII, IX, or XI present if lupus anticoagulant is negative. APTT = activated partial thromboplastin time.

Anticoagulant assay was performed for this patient before surgery. Surprisingly, this patient had normal values for PT, TT, and fibrinogen, but had isolated prolonged APTT value (20 seconds longer than normal). The clinicians questioned the correctness of this result as the patient showed normal APTT in a routine laboratory of the local hospital (Table 2), and the patient didn’t have any abnormal bleeding or thrombotic episodes. For the smooth operation, analysis of the causes of isolated prolonged APTT must be carried out by the laboratory specialist. After excluding other causes (elevated hematocrit, contamination by anticoagulants, prolonged specimen retention time, and taking anticoagulants), we conducted mixing studies according to the detection process in Fig. 1.^[6]^ Based on our results, the ICA for immediate mixing was <15%, indicating no coagulation factor deficiency. Time incubation mixing highlighted that the abnormally prolonged APTT was caused by antiphospholipid antibodies (Table 1).

**Table 2:** Tests for antiphospholipid antibodies, thyroid hormones, and platelet count monitoring after admission.

Indicators (Unit)	Test result	Reference value
ACA-IgG (GPL/mL)	<20.0 (−)	<20.0 (−)
ACA-IgM (MPL/mL)	<20.0 (−)	<20.0 (−)
Anti-β2 GPI-IgG (SGU/mL)	<20.0 (−)	<20.0 (−)
Anti-β2 GPI-IgM (SMU/mL)	<20.0 (−)	<20.0 (−)
LA dRVVT normalized ratio	2.36	<1.2
Platelet count (∗10^9^/L)	236	100–300
FT3, pmol/L	3.51	2.43–6.01
FT4, pmol/L	14.51	9.01–19.05
TSH, mIU/L	2.78	0.35–4.94
TG, μg/L	<0.04	1.40–78.00
TGAb, IU/mL	231.06	<4.11
TPOAb, IU/mL	60.05	<5.61

ACA = anticardiolipin antibodies, FT3 = free triiodothyronine, FT4 = free tetraiodothyronine, GPI = glycoprotein I, LA = lupus anticoagulant, TG = thyroglobulin, TGAb = antithyroglobulin antibodies, TPOAb = thyroid peroxidase antibodies.

Subsequently, we tested for antiphospholipid antibodies that could cause prolongation of any phospholipid-dependent coagulation tests. Table 2 showed that anticardiolipin antibodies and anti-β2 glycoprotein I antibodies were negative, while LA was strongly positive (dRVVT normalized ratio: 2.36). Accordingly, we determined that the cause of a prolonged APTT in this patient was the interference of LA. Platelet count was normal, indicating that the patient had a tendency to in-vivo thrombosis rather than a bleeding diathesis. At last, we communicated with the clinician and suggested that there was no specific treatment suggestion before the operation because the patient had no abnormal bleeding. This thyroid cancer patient had an uneventful surgery and was discharged after a week. Unfortunately, the patient did not come to our hospital again, we could not confirm whether she developed antiphospholipid syndrome due to the persistently positive antiphospholipid antibodies.

## 3 Discussion

Clotting time is a routine preoperative examination for each patient before surgical intervention. Our case showed a normal PT and TT, prolonged APTT of 65.3 seconds (reference range: 30–45 seconds), whereas, 2 days before admission into our hospital, she had been tested for these items normally at a local hospital. The further detailed examination of factors affecting APTT prolongation was executed in our laboratory. Generally, isolated prolonged APTT is often observed in the following cases: patients treated with anticoagulant medications such as heparin or agatroban, patients lacking specific coagulation factors, and patients with LA.^[7]^ However, this patient did not use any anticoagulants and had no liver disease or bleeding symptoms. Mixing studies was conducted to determine whether abnormality was secondary to coagulation factor deficiency or an inhibitor. In this study, 2 types of mixing studies were performed: immediate mixing study and time incubation mixing study. APTT correction was not evident in our case, instead, an inhibitor was present. As we expected, LA was positive according to the screening and confirmatory tests with the dilute Rusell viper venom time. Finally, it's clear that the LA was responsible for prolonged APTT in this case.

Lupus anticoagulants are a group of immunoglobulins that bind directly to negatively charged phospholipids or phospholipid protein complexes.^[8]^ Patients with persistent LA are considered at high risk of thrombosis and recurrence, as well as being a danger signal for thrombotic disorders and certain autoimmune diseases.^[3,9]^ The main feature of lupus anticoagulants is their ability to prolong phospholipid-dependent clotting time in vitro, mainly the APTT, but mixing studies do not lead to correction or normalization of APTT.^[10]^ However, since the different types of APTT reagent activators or different phospholipid concentrations used for commercial testing, there are considerable differences in sensitivity to coagulation factor deficiency, heparin, and LA.^[11–13]^ The most common commercially available activators are silica and ellagic acid.^[14]^ The different APTT results between her local hospital and our hospital may be caused by large different sensitivity of the reagents used. Therefore, it is critical to select highly sensitive reagents or to use multiple reagents for mutual validation.^[15]^

Hashimoto thyroiditis is considered to be an autoimmune disease that is associated with a variety of autoimmune phenomena. From Hashimoto thyroiditis to thyroid cancer, a series of changes may be encountered. To our knowledge, the association of LA with Hashimoto thyroiditis has not been systematically studied previously, only one case reported.^[16]^ We speculate that the presence of antiphospholipid antibodies in Hashimoto thyroiditis patients is not surprising given the association of the disease with autoimmune and hematologic disorders. Future studies investigating whether LA persists in Hashimoto thyroiditis patients are warranted.

## 4 Conclusion

Isolated prolongation of APTT due to underlying LA may lead to unnecessary delayed surgical intervention or invasive procedure. The root causes of prolonged APTT need to be identified to ensure the correct therapeutic approach, otherwise severe cases may be fatal.

## Acknowledgments

The authors appreciate everyone in the Department of Clinical Hematology Laboratory and Thyroid surgery, the Second Affiliated Hospital, Zhejiang University School of Medicine.

## Author contributions

**Conceptualization:** Yuhong Zhong, Biyong Yan.

**Data curation:** Yuhong Zhong, Zhongqi Yu.

**Formal analysis:** Yuhong Zhong, Lin Wang.

**Investigation:** Biyong Yan, Zhongqi Yu, Lin Wang.

**Methodology:** Yuhong Zhong.

**Validation:** Biyong Yan, Zhongqi Yu.

**Writing – original draft:** Yuhong Zhong, Biyong Yan.

**Writing – review & editing:** Yuhong Zhong, Lin Wang.
